# Characterization of Indoxacarb Resistance in the Fall Armyworm: Selection, Inheritance, Cross-Resistance, Possible Biochemical Mechanisms, and Fitness Costs

**DOI:** 10.3390/biology11121718

**Published:** 2022-11-27

**Authors:** Muhammad Hafeez, Xiaowei Li, Farman Ullah, Zhijun Zhang, Jinming Zhang, Jun Huang, Limin Chen, Junaid Ali Siddiqui, Xiaoyun Ren, Shuxing Zhou, Muhammad Imran, Mohammed A. Assiri, Myron P. Zalucki, Yonggen Lou, Yaobin Lu

**Affiliations:** 1 State Key Laboratory of Rice Biology, Ministry of Agriculture Key Lab of Molecular Biology of Crop Pathogens and Insects, Institute of Insect Sciences, Zhejiang University, Hangzhou 310058, China; 2 State Key Laboratory for Managing Biotic and Chemical Threats to the Quality and Safety of Agro-Products, Institute of Plant Protection and Microbiology, Zhejiang Academy of Agricultural Sciences, Hangzhou 310021, China; 3 Department of Plant Biosecurity, College of Plant Protection, China Agricultural University, Beijing 100083, China; 4 Integrated Plant Protection Center, Lishui Academy of Agricultural and Forestry Sciences, Lishui 323000, China; 5 College of Agriculture, College of Tobacco Science, Guizhou University, Guiyang 550025, China; 6 Department of Chemistry, Faculty of Science, King Khalid University, P.O. Box 9004, Abha 61413, Saudi Arabia; 7 School of Biological Sciences, The University of Queensland, Brisbane, QLD 4072, Australia

**Keywords:** *Spodoptera frugiperda*, indoxacarb selection, cross-resistance, inheritance of resistance, fitness cost

## Abstract

**Simple Summary:**

The fall armyworm (FAW), *Spodoptera frugiperda* (J.E. Smith), is a voracious insect pest native to the Western Hemisphere, particularly in South America. The polyphagous fall armyworm feeds on more than 350 plants in such families, including Poaceae, Asteraceae, and Fabaceae. Transgenic plants that express *Bacillus thuringiensis* proteins (Bt plants) and synthetic insecticides are the main tactics to control *Spodoptera frugiperda*, although widespread usage of synthetic chemicals has resulted in the emergence of resistance. We assessed cross-resistance, resistance mechanism, and fitness costs based on the life history traits of *Spodoptera frugiperda*. Results indicated that after 24 generations of selection, the resistance to indoxacarb was increased by 472.67-fold as compared to the Ind-UNSEL. Significantly longer developmental time of larvae extended pupal duration, shorter adult longevity, and lower fecundity were observed in Ind-SEL as compared with the Ind-UNSEL population. Butoxide synergist increased susceptibility to indoxacarb, indicating that P450 enzymes may be involved in indoxacarb resistance. Therefore, it is crucial that we comprehend how insecticides work and how resistance develops in order to develop techniques for managing resistance. These data are valuable to understand the indoxacarb resistance mechanism and provides vital information for scientific-based guidance of pest management decisions.

**Abstract:**

The fall armyworm, *Spodoptera frugiperda* (J.E. Smith), is a voracious insect pest that is difficult to control due to resistance to insecticides and Bt proteins. We assessed cross-resistance, resistance mechanism, and fitness costs based on the life history traits of *S. frugiperda*. We established an *S. frugiperda* strain selected for resistance to indoxacarb (Ind-SEL) from a field-collected population and an unselected strain, Ind-UNSEL. Results indicated that after 24 generations of selection, the resistance to indoxacarb was increased by 472.67-fold as compared to the Ind-UNSEL. There was high cross-resistance to deltamethrin (31.23-fold) with very low or negligible cross-resistance to chlorantraniliprole, emamectin benzoate, and/or methoxyfenozide in the Ind-SEL population. Butoxide synergist increased susceptibility to indoxacarb, indicating that P450 enzymes may be involved in indoxacarb resistance. Significantly longer developmental time of larvae extended pupal duration, shorter adult longevity, and lower fecundity were observed in Ind-SEL as compared with the Ind-UNSEL population. The Net reproductive rate (*R*_0_) was the only growth parameter that differs between crosses of Ind-SEL♂ × Ind-UNSEL♀ (176 ± 46) and Ind-SEL♀ × Ind-UNSEL♂ (328 ± 57). On the other hand, all population growth parameters differ between Ind-SEL and Ind-UNSEL strains. Our work contributes to the growing body of research that demonstrates the importance of strain genetics in fitness cost experiments and helps resistance management programs make decisions.

## 1. Introduction

The fall armyworm (FAW), *Spodoptera frugiperda* (J.E. Smith), is a voracious insect pest native to the Western Hemisphere, particularly in South America [1]. It is one of the most rapidly spreading and highly invasive pests of maize across Africa and Asia [2,3,4,5]. *S. frugiperda* has become a pest species because of its biological characteristics such as polyphagy, concealed larval feeding habits, high reproductive capacity, adult dispersion, and multiple generations per year [3,4,5]. The polyphagous fall armyworm feeds on more than 350 plants in such families, including Poaceae, Asteraceae, and Fabaceae [6]. The FAW has two haplotypes that have been recognized for a long time: the “rice strain” (R strain), which prefers to eat rice and grasses, and the “corn strain” (C strain), which prefers to eat maize and sorghum [7,8,9]. It is very important to control *S. frugiperda* infestations and spread because it causes severe economic loss to several economically important crop plants and threatens global food security and the livelihoods of many households [2,5]. Currently, *S. frugiperda* represents a serious problem to maize crops in China and elsewhere in South Asia [4,10]. 

Transgenic plants that express *Bacillus thuringiensis* proteins (Bt plants) and synthetic insecticides are the main tactics to control FAW, although widespread usage of synthetic chemicals has resulted in the emergence of resistance [11,12,13,14]. Unfortunately, the foliar application of chemical insecticides against the *S. frugiperda* population in Bt and non-Bt maize crops has low control efficacy [13]. This may be due to the feeding behavior of *S. frugiperda* larvae which stay inside the maize whorl, thus reducing insecticide contact. In addition, Long-term use of chemical pesticides in the field poses serious risks to the environment due to contamination and causes the death of natural enemies, which often leads to pest resurgence, and inevitably has led to insecticide-resistant in different insects population [11,15,16,17,18,19,20]. Previously, some studies have shown that multiple field populations of *S. frugiperda* have developed high-level resistance as well as broad cross-resistance to diverse groups of synthetic insecticides, including pyrethroids, organophosphate, carbamate, chlorantraniliprole, abamectin, emamectin benzoate, lufenuron and spinosad [11,12,13,14,18,19]. In addition, widespread areas of Bt-crops without growing refuge in some mainly tropical countries have increased the evolution of resistance problems to Bt proteins in *S. frugiperda* populations [21,22,23]. The effort to control this pest is becoming exceedingly challenging all over the world.

Indoxacarb is a new oxadiazine insecticide with significant toxicity against a variety of lepidopteran, coleopteran, and sucking insect pests in agricultural as well as urban contexts [24]. Insect esterases or amidases can convert indoxacarb to an N-decarbomethoxylated metabolite (DCJW), a more potent sodium channel blocker than indoxacarb, which causes the target pest species to become paralyzed and die [25,26]. Indoxacarb is highly active when ingested, but there have been few reports of contact activity when applied topically [24,26,27,28]. Indoxacarb is a potent novel insecticide for crop protection because to its safety for humans and non-target organisms, superior environmental and residual qualities, broad spectrum, and quick reduction in insect feeding [24]. However, numerous studies have indicated that a number of insects have recently evolved resistance to indoxacarb due to its widespread use, including *S. litura* [29], such as *C. rosaceana* [30], *M. domestica* [31], *S. exigua* [32], *P. xylostella* [33] and *H. armigera* [34] have developed a significant level of resistance. Furthermore, resistance to several Bt maize products expressing Cry1F and Cry1Ab proteins has been reported in the field, increasing the use of chemical insecticides against *S. frugiperda* in maize [13,35]. The detoxification enzymes P450, esterase, and glutathione S-transferase (GST) are involved in the resistance to indoxacarb [33,34,36]. Additionally, novel sodium channel mutations (F1845Y and V1848I) have been reported to be associated with resistance to indoxacarb in *P. xylostella* and *T. absoluta* [33,37]. However, indoxacarb resistance is currently at a relatively low level in *S. frugiperda* populations, although the pest has been subjected to indoxacarb selection pressure. Insecticide resistance management in insect pests is a global challenge for entomologists. However, if resistance to a novel insecticide can be monitored and predicted, a proactive resistance management program can be established to reduce the risk of resistance [23,38,39]. Laboratory selection experiments can provide important insect resistance data [14,40]. Therefore, it is crucial that we comprehend how insecticides work and how resistance develops in order to develop techniques for managing resistance. Additionally, figuring out the molecular basis behind pesticide resistance may open up new possibilities for the creation of cutting-edge tactics for controlling insect pests. However, indoxacarb resistance mechanism, inheritance, and resistance-associated fitness costs in *S. frugiperda* have not been documented to date.

Understanding the resistance mechanism and resistance-associated fitness costs is essential as they directly affect the rate of resistance evolution in the field population and play significant roles in an insect resistance management (IRM) strategy [41,42]. Moreover, evaluating the fitness costs associated with resistance can aid in determining if vulnerability can be regained in the context of selection pressure [43,44,45]. In this study, to understand the potential mechanism of the fast-evolved resistance to indoxacarb, the inheritance of resistance and resistance-associated fitness costs in the *S. frugiperda* strain from a field population was evaluated. These data are valuable to understand the indoxacarb resistance mechanism and provides vital information for scientific-based guidance of pest management decisions.

## 2. Materials and Methods

### 2.1. Collection and Breeding of Insects 

The larvae of the field population of FAW were originally collected from two different corn fields in Ping Hu County (Latitude: 30.705° N, Longitude: 121.118° E) Zhejiang Province in 2019, denoted as (PHZ19) and maintained on an artificial diet under control conditions at 26 ± 2 °C, 65 ± 5% relative humidity (RH), 14:10 h (light:dark, L:D) photoperiod until adults emerged. The newly emerged adults were paced in mating cages, according to Hafeez et al. (2021) [4], and fed a 10% sugar solution as a food source.

### 2.2. Toxicological Bioassays 

The tested insecticide, indoxacarb (15% commercial formulation), was purchased from Mesa Tech Co., Ltd. (Beijing China). Using the diet-incorporation technique described by [46], a preliminary bioassay was carried out using second-instar larvae from the G2 generation with various concentrations of indoxacarb. The field-collected population (PHZ19) was reared on an artificial diet under laboratory conditions for one generation before bioassays. Insecticide concentrations were obtained from freshly prepared stock solution following serial dilutions for tested insecticide. The surfactant, Triton X–100, was used at 0.1% to each serial concentration to achieve a uniform mixture of the insecticide solution in the diet. Six concentrations of tested insecticide were used to determine the LC_50_ concentration with three replications per concentration. Each serial concentration was thoroughly mixed into the artificial diet before the agar solidified (40–45 °C) using the method developed [17]. After cooling, the diet was cut into small cubes and transferred into new sterile transparent plastic cups (3 cm diameter, 3.5 cm height). The artificial diet without insecticide was used as a standard control treatment. A total of 90 two-day-old second-instar larvae were used per concentration, with three replicates established for each concentration (30 larvae per replicate). Similarly, the control was prepared using 0.1% Triton X–100 in distilled water. Six concentrations of indoxacarb (0.125–400 μg mL^−1^ of diet) were used for the Ind-UNSEL and Ind-SEL strains. All were kept in a climate control chamber, as described above. Mortality was evaluated 72 h after exposure to indoxacarb. Larvae that did not move after being touched with a fine paintbrush were deemed dead.

### 2.3. Protocol for Indoxacarb Resistance Selection

A preliminary bioassay of indoxacarb was performed with the field population (PHZ19) to determine the lethal concentration (LC_50_) required for the selection of *S. frugiperda* with indoxacarb so that sufficient survivors were left for the next generation. Second instar larvae (one day old) were selected to be exposed continuously to lethal concentrations (LC_50_) of indoxacarb insecticide from G1 to G24 (Table 1). The diet incorporation method was used for the selection of bioassays. For each selection, an indoxacarb-treated diet was cut into small pieces (1 g) and kept in Petri dishes. The treated population in Petri dishes was placed in the laboratory under the conditions described above. Mortality data were taken after 72 h exposure to indoxacarb in each selection. After 72 h post-exposure, larvae that presented a survival rate of more than 50% were considered positive for resistance. Survivors of each selection were reared on a diet without exposure to indoxacarb to obtain the next progeny for subsequent indoxacarb selection. 

### 2.4. Inheritance of Resistance

In order to assess the inheritance of resistance to indoxacarb in *S. frugiperda*, one-day-old second instar larvae from Ind-UNSEL and reciprocal crosses were used in a diet-incorporation method in small transparent Petri-dishes. Six to seven serial concentrations of indoxacarb were prepared in distilled water and thoroughly mixed in a freshly prepared artificial diet as described above. The surfactant, Triton X–100 (www.biofroxx.com) accessed on 14 March 2021, was used at 0.1% for each serial concentration to achieve a uniform mixture of the insecticide solution in the diet. The control was prepared using 0.1% Triton X–100 in distilled water. After cooling the diet, 90 s-instar larvae (one day old) per concentration with triplicate (30 larvae in each replicate) were transferred into each Petri Plate and shifted in a climatic chamber at standard conditions. 72 h after exposure to insecticide, the larval mortality was assessed. When stroked with a delicate paintbrush, larvae that did not move were assumed to be dead. Resistance ratios were calculated by dividing the LC_50_ values of the Ind-SEL or reciprocal cross by the corresponding parameter for the Ind-UNSEL strain, as described by [47]

### 2.5. The Degree of Dominance (D)

In order to determine the dominance of resistance, individual pupa from the Ind-SEL and Ind-UNSEL population was placed in transparent cups (50 mL). A reciprocal cross between Ind-SEL♂ × Ind-UNSEL ♀ and Ind-SEL♀ × Ind-UNSEL♂ populations were made (20 couples per cross) after adult emergence. The F1 offspring from each reciprocal cross was maintained on an artificial diet [48]. Second-instar larvae (one day old) larvae from Ind-SEL, Ind-UNSEL, and reciprocal crosses were then exposed to the concentration-response bioassays as described above. The degree of dominance (D) of indoxacarb resistance was estimated using the equation defined by [49]

*D_ML_* = (*ML_RS_*-*ML_SS_*)/(*ML_RR_*-*ML_SS_*) (1)

where *M_RR_*, *M_SS,_* and *M_RS_* are the mortalities level of Ind-UNSEL, Ind-SEL, and reciprocal crosses, respectively, in different concentrations of indoxacarb. *D_ML_* levels near 0 indicate effective recessive resistance, whereas values close to 1 indicate effective dominant resistance, and intermediate values imply effectively incomplete resistance.

### 2.6. Cross-Resistance of Indoxacarb to Insecticides in S. frugiperda

Late second-instar larvae from the Ind-SEL and Ind-UNSEL strains were treated with several groups of pesticides using the diet-incorporation technique, as previously mentioned, to assess patterns of cross-resistance. The different groups of insecticides indoxacarb 15%, deltamethrin 25EC, chlorfenapyr 10%, cholorantraniliprole 5%, methoxyfenozide 240 SC, spinosad 5%, and emamectin benzoate 2% were tested. Concentration–mortality data were submitted to the same procedures described above in toxicity bioassays.

### 2.7. Synergism Bioassay

Bioassays with piperonyl butoxide (PBO), triphenyl phosphate (TPP), and diethyl maleate (DEM) as synergists (Aladdin Bio-chem Technology Co., Ltd., Shanghai, China) were carried out to assess the possible metabolic resistance to indoxacarb in *S. frugiperda* using the method described [17]. The stock solutions at the concentration of PBO; 50 mg/L, TPP; 50 mg/L, and DEM; 100 mg/L, were diluted in acetone (99.5% purity), respectively. Larvae were then treated with 1 μL of acetone/PBO solution as above; PBO (50 μg μL^−1^) using a hand applicator. The larvae were put into a transparent plastic cup with an indoxacarb-treated diet at various concentrations and a control diet that was subjected to 1 μL acetone alone for 72 h after exposure with synergists and without synergists for 2 h. All treatments were kept at a constant condition, as explained above. After 72 h exposure to the test insecticide, the larval mortality was evaluated.

### 2.8. Fitness Cost

In order to evaluate the fitness costs associated with indoxacarb resistance in *S. frugiperda*, 50 eggs were selected from Ind-SEL, Ind-UNSEL, and reciprocal crosses (Indo-SE♂ × Ind-UNSEL♀ and Ind-SEL♀ × Ind-UNSEL♂) and hatched neonates were transferred separately into small transparent plastic cups containing an artificial diet. All life history parameters such as developmental period of eggs, larval and pupae, adults (male and female) life span, and daily observations were used to calculate total fecundity (eggs per female), oviposition period, total developmental duration and survival (egg to adult), oviposition duration. There were 20 couples per treatment maintained in plastic mating cages that were 23 cm height by 10 cm wide, internally ornamented with the white paper that served as the oviposition substrate and covered at the top with a lid to measure the female fecundity. All Adults pairs were provided with a 10% honey solution as food.

The life-history data of Ind-SEL, Ind-UNSEL, and reciprocal crosses (Indo-SE♂ × Ind-UNSEL♀ and Ind-SEL♀ × Ind-UNSEL♂) were subjected to the computer-based program software (TWOSEX-MSChart) [50] and analyzed using age-stage two-sex life table theory [51,52]. The life table parameters such as age-stage survival rate (*s_xj_*), age-specific survival rate (*l_x_*), age-specific fecundity (*m_x_*), age-stage life expectancy (*e_xj_*), and age-stage reproductive value (*v_xj_*), respectively, were estimated (where *x* is the age and *j* are the stages of insect). The population parameters, including the intrinsic rate of increase (*r*), finite rate of increase (*λ*), net reproductive rate (*R_0_*), and mean generation time (*T*), was also estimated. The life expectancy (*e_xj_*) and the reproductive value (*v_xj_*) were determined according to [53,54,55]. The standard bootstrap method was used with 100,000 resampling to calculate the variance as well as standard errors for biological and population growth parameters [56,57] Paired-bootstrap-test at a 5% significance level based on the confidence interval of differences was applied to analyze differences among treatments using TWOSEX-MSChart computer program.

### 2.9. Statistical Analysis

Toxicity bioassay data were used to calculate the lethal (LC_50_) concentrations of indoxacarb by using Probit analysis [58] in PoloPlus 2.0 [59]. Data related to the life table was analyzed using the TWOSEX-MSChart computer program. All life table-related graphs were created using Sigma plot 12.5.

## 3. Results

### 3.1. Selection of S. frugiperda Resistant to Indoxacarb 

The LC_50_ value of indoxacarb against second-instar larvae of the field-collected population of *S. frugiperda* was 0.674 μg mL^−1^ (Table 1), and with selection, the LC_50_ value of Ind-SEL from generation G2 to G24 increased from 0.787 to 201.83 μg mL^−1^. Our Ind-UNSEL population had an LC_50_ of 0.427 μg mL^−1^ indicating *S. frugiperda* showed a maximum resistance ratio (RR) to indoxacarb of 299.45-fold after 24 generations (Table 1). 

### 3.2. Genetic Inheritance of Resistance to Indoxacarb

In resistance characterization studies, the indoxacarb concentrations needed for the assessment of LC_50_ values varied among Ind-SEL, Ind-UNSEL, and reciprocal strain crosses (Table 2). The resistance ratio of indoxacarb in the reciprocal cross, Indo-SE♂ × Ind-UNSEL♀ and Ind-SEL♀ × Ind-UNSEL♂ were 7.072- and 6.42-fold as compared with Ind-UNSEL. LC_50_ of reciprocal crosses were significantly different due to the overlapping of 95% CIs, demonstrating that indoxacarb resistance was not autosomal. Rather sex linkage or maternal effects were present in the tested *S. frugiperda* populations.

### 3.3. Dominance of Resistance

**Figure 1:**
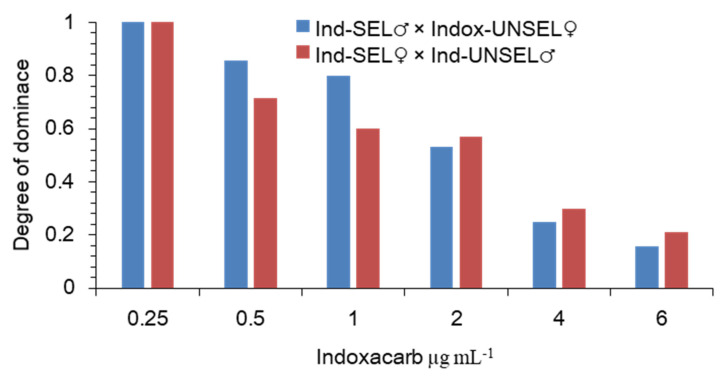
Degree of the dominance of resistance to indoxacarb in *S. frugiperda* as a function of indoxacarb concentration.

The degree of the dominance was calculated following the method of Bourguet et al. (2000) showed that the dominance values decreased as the indoxacarb concentrations increased. However, higher levels of dominance occurred at lower concentrations. The level of dominance was lower than 0.5 at the concentration of 3 μg-mL^−1^, which indicated an incompletely recessive dominance when the *S. frugiperda* larvae were exposed to this insecticide (Figure 1). Resistance can be defined as a dominant trait when (*D_ML_* = 1). Using the method explained by the degree of dominance decreased with increasing concentration of indoxacarb, supporting an incompletely recessive inheritance at the higher concentrations (Figure 1).

### 3.4. Susceptibility of and Cross-Resistance of Ind-SEL and Ind-UNSEL Genotypes of S. frugiperda to Different Insecticides

The bioassays with deltamethrin, chlorfenapyr, cholorantraniliprole, methoxyfenozide, spinosad, and emamectin benzoate performed on the Ind-SEL population (G24) showed that the selection of *S. frugiperda* with indoxacarb induced very low cross-resistance to chlorfenapyr (RR = 3.24-fold; LC_50_ = 3.92 µg mL^−1^) and cholorantraniliprole (RR = 1.89-fold; LC_50_ = 1.09 µg mL^−1^), methoxyfenozide (RR = 1.59-fold; LC_50_ = 2.32 µg mL^−1^), spinosad (RR = 2.65-fold; LC_50_ = 1.27 µg mL^−1^) and emamectin benzoate (RR = 1.98-fold; LC_50_ = 0.89 µg mL^−1^) but a high level of cross-resistance to deltamethrin (RR = 31.23-fold; LC_50_ = 25.3 µg mL^−1^) when compared to Ind-UNSEL population (Table 3).

### 3.5. Synergism of PBO, TPP and DEM

The synergism of PBO, TPP, and DEM was tested on indoxacarb in Ind-SEL (G24) and Ind-UNSEL (G24) (Table 4). Two synergists, PBO, significantly synergized the toxicity of indoxacarb in Ind-SEL (95% CI did not overlap) with resistance ratios of 3.22-fold, respectively. In contrast to this, DEM and TPP did not show any significant synergistic effect in Ind-SEL (95% CI overlap) (Table 4). These results suggest the two detoxification enzymes, mono-oxygenases, might play an important role in detoxifying indoxacarb and the development of resistance in *S. frugiperda*.

### 3.6. Fitness Costs with Distant-Related Genetic Backgrounds of S. frugiperda Strains

The development time for the egg stage (3.0 ± 0.0 d) did not differ significantly among strains, but there were significant differences among different larval stages with extended developmental time observed in the Ind-SEL strain. The total larval developmental time of the Ind-SEL strain was approximately (23.34 ± 0.33 d) as compared to the Ind-SEL♂ × Ind-UNSEL♀ (21.81 ± 0.18 d), Ind-SEL♀ × Ind-UNSEL♂ (22.32 ± 0.13 d) and the Ind-UNSEL (19.77 ± 0.13 d) (Table 5). The pupal developmental duration of the Ind-SEL strain (9.26 ± 0.66 d) was significantly higher as compared to the Ind-SEL♀ × Ind-UNSEL♂ (8.19 ± 0.66 d) and Ind-UNSEL strain (7.5 ± 0.08 d) respectively (Table 5).

Longevity differed for female and male adults, with shorter longevity of female adults in the Ind-SEL (11.58 ± 0.58 d) and Ind-SEL♂ × Ind-UNSEL♀ (11.25 ± 0.3 d) as compared to Ind-SEL♀ + Ind-UNSEL♂ (12.6 ± 0.17 d) and Ind-UNSEL (12.91 ± 0.28 d) while no difference was noted between and Ind-SEL and Ind-SEL♂ × Ind-UNSEL♀. Whereas male adult longevity was significantly shorter in Ind-SEL♀ × Ind-UNSEL♂ (7.63 ± 0.99 d) as compared to other strains (Table 6). Similarly, significantly shorter longevity of adults was noted for Ind-SEL (9.58 ± 0.7 d) and the Ind-SEL♂ × Ind-UNSEL♀ (9.51 ± 0.54 d) as compared to the Ind-SEL♀ × Ind-UNSEL♂ and Ind-UNSEL while no significant difference was observed between Ind-SEL and Ind-SEL♂ × Ind-UNSEL♀ respectively (Table 6). The total pre-oviposition period (TPOP) was comparatively extended in the Ind-SEL strain (37.45 ± 0.56 d) followed by Ind-SEL♀ × Ind-UNSEL♂ (35.45 ± 0.37 d) and the Ind-SEL♂ × Ind-UNSEL♀ (33.75 ± 0.57 d) as compared to Ind-UNSEL (30.74 ± 0.18 d) (Table 6). A similar trend was noted for mean generation time in all strains. The total number of eggs produced per female was significant differences among strains. A significantly lower number of eggs per female was observed in the Ind-SEL (612.92 ± 68.02) and the Ind-SEL♂ × Ind-UNSEL♀ (732.0 ± 53.42) strain as compared to Ind-SEL♀ × Ind-UNSEL♂ and Ind-UNSEL (Table 6). The population parameters indicated significant differences among strains. The intrinsic rate of increase (*r*), net reproductive rate (*R*_0_), and Finite rate of population increase were significantly lowered in the Ind-SEL strain, followed by Ind-SEL♂ × Ind-UNSEL♀ and Ind-SEL♀ × Ind-UNSEL♂ as compared to Ind-UNSEL. The mean length of a generation (T) was significantly higher for the Ind-SEL strain (*T* = 40.65 ± 0.57 d) followed by Ind-SEL♀ × Ind-UNSEL♂ (38.61 ± 0.32 d) as compared to Ind-UNSEL (34.1 ± 0.23 d) and there was no statistical difference between Ind-SEL and Ind-SEL♀ × Ind-UNSEL♂ strains (Table 7).

### 3.7. Survival Rate of S. frugiperda Calculated by Two-Sex Life Table Analysis

**Figure 2:**
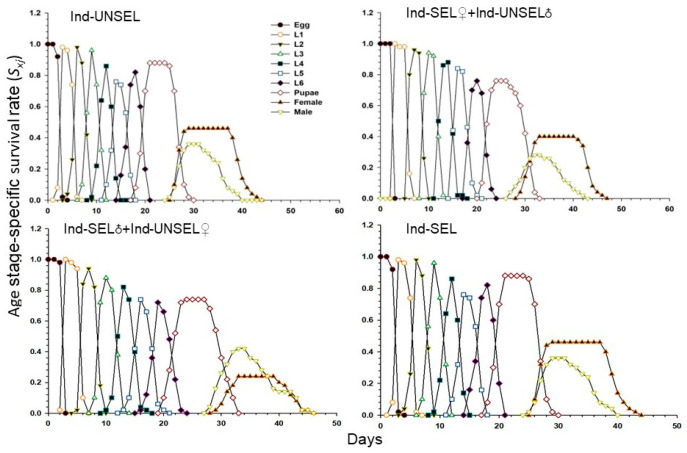
Age-stage specific survival rate (*s_xj_*) of Ind-UNSEL, Ind-SEL♀ × Ind-UNSEL♂, Ind-SEL♂ × Ind-UNSEL♀ and Ind-SEL population of *S. frugiperda.*

Survival rate (*S*_xj_) of the Ind-UNSEL, Ind-SEL♀ × Ind-UNSEL♂, Ind-SEL♂ × Ind-UNSEL♀, and Ind-SEL are shown in (Figure 2). The values differed significantly across the different developmental stages, suggesting that the growth rates differed among the individuals. The survival curves of different age stages of *S. frugiperda* larvae overlap, and larvae completed development at 21 days in Ind-SEL and Ind-UNSEL, compared with the Ind-SEL♀ × Ind-UNSEL♂ (24 days) and Ind-SEL♂ × Ind-UNSEL♀ (23 days) strains (Figure 2). However, there was shorter survival of adults in the Ind-UNSEL and Ind-SEL♀ x Ind-UNSEL♂ (20 days for both) as compared to the Ind-SEL♂ × Ind-UNSEL♀ and Ind-SEL. Similarly, the age stage-survival rate of males and females of *S. frugiperda* from egg to adult were in Ind-UNSEL (0.27 and 0.45), Ind-SEL♀ × Ind-UNSEL♂ (0.24 and 0.4), Ind-SEL♂ × Ind-UNSEL♀ (0.4 and 0.22) and Ind-SEL (0.42 and 0.28) respectively, (Figure 2).

### 3.8. Population Survival Rate and Fecundity of S. frugiperda

**Figure 3:**
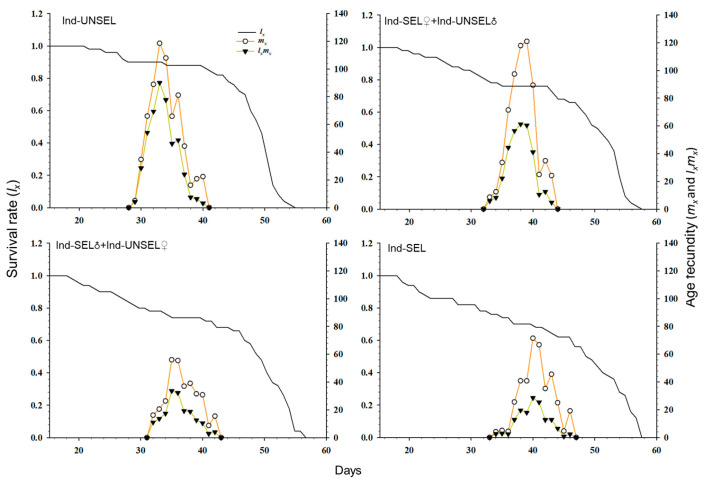
Age-specific survival rate (*l_x_*), age-specific fecundity (*m_x_*), and net maternity (*l_x_m_x_*) of Ind-UNSEL, Ind-SEL♀ × Ind-UNSEL♂, Ind-SEL♂ × Ind-UNSEL♀ and Ind-SEL population of *S. frugiperda.*

The age-specific survival rate (*s_xj_*) and fecundity of *S. frugiperda* (Figure 3): *l_x_* and *l_x_m_x_* on Ind-SEL (56 days 125 days) showed a downward trend as compared to Ind-UNSEL (55 days, 200 days). Thus, the results indicated that the selection pressure of insecticide was not in favor of the development and reproduction of *S. frugiperda*. Furthermore, the deviations in the fecundity curve of *S. frugiperda* were advocated that the emergence and oviposition did not happen at specific ages and times, respectively (Figure 3).

### 3.9. Reproduction Value and Life Expectancy of S. frugiperda

**Figure 4:**
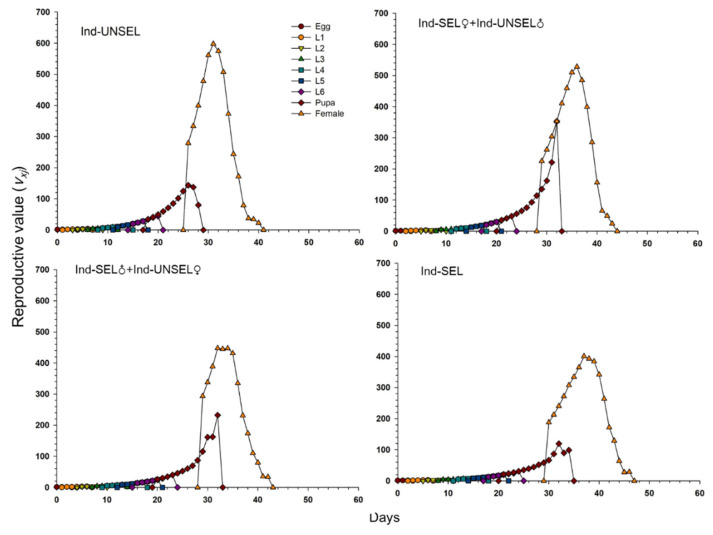
Age-stage specific life expectancy (*e_xj_*) of Ind-UNSEL, Ind-SEL♀ × Ind-UNSEL♂, Ind-SEL♂ × Ind-UNSEL♀ and Ind-SEL population of *S. frugiperda.*

**Figure 5:**
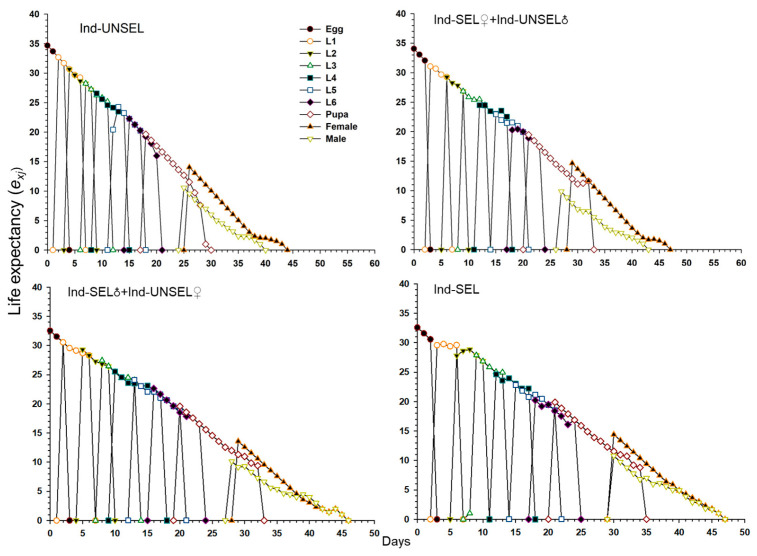
Age-Stage specific reproductive value (*v_xj_*) of Ind-UNSEL, Ind-SEL♀ × Ind-UNSEL♂, Ind-SEL♂ × Ind-UNSEL♀ and Ind-SEL population of *S. frugiperda.*

Significantly lower reproductive value (*v_xj_*) of *S. frugiperda* on Ind-UNSEL (1.197), Ind-SEL♀ × Ind-UNSEL♂ (1.162), Ind-SEL♂ × Ind-UNSEL♀ (1.151) and Ind-SEL (51.13) at age zero (v0, 1), respectively, which were close to λ (Figure 4). The peak value of the *v_xj_* curve of all strains exhibited an upward with increasing trend age and developmental stage, with the maximum value at 31 days on Ind-SEL (609.7) and at 36 days on Ind-SEL♀ × Ind-UNSEL♂ (539.769) as compared to other strains (Figure 4). Furthermore, the life expectancy value (*e_xj_*) of *S. frugiperda* on all strains indicated a decreasing trend, with significantly highest average longevity values on Ind-UNSEL (35 days), Ind-SEL♀ × Ind-UNSEL♂ (34 days), Ind-SEL♂ × Ind-UNSEL♀ (33 days) and Ind-SEL of (32 days), respectively (Figure 5). The *e_xj_* value of *S. frugiperda* was lower on Ind-SEL and Ind-SEL♂ × Ind-UNSEL♀ than on Ind-SEL♀ × Ind-UNSEL♂ and Ind-UNSEL in the first 9 days, but the trend was reversed afterward, representing that *S. frugiperda* developed more slowly on Ind-SEL and Ind-SEL♂ × Ind-UNSEL♀ (Figure 5).

## 4. Discussion

The selection pressure induced by the indiscriminate use of pesticides has resulted in the dramatic evolution of insecticide resistance in insect pests. Indoxacarb belongs to the novel oxadiazine group with wide-spectrum insecticidal activity against several lepidopteran species as well as some other groups of insect pests such as homopteran and coleopteran and has a low toxicity profile for non-target organisms [24,60]. Numerous insect species have been used to investigate the mechanisms underlying indoxacarb resistance. Two mutations (F1845Y and V1848I) have been found in indoxacarb-resistant populations of two pest species, *Plutella xylostella* [61] and *Tuta absoluta* [38]. Gao et al. [32] identified one point mutation (L1014F) in indoxacarb selected strain of *S. exigua,* whether L1014F mutation in *S. exigua* is associated with indoxacarb resistance or not the functional verification is needed by gene editing or electrophysiology. Similarly, Samantsidis et al. (2019) [62] and Wang et al. (2022) [63] found that only F1845Y and V1848I mutations had been proven to confer resistance to indoxacarb in *Plutella xylostella* and *Drosophila*. In our study, we showed a significant selection response to indoxacarb in a field-collected population of *S. frugiperda.* Following continuous laboratory selection for 24 generations, a field-collected population Ind-SEL of *S. frugiperda* exhibited a very high level of resistance (472.67-fold) to indoxacarb as compared to the Ind-UNSEL population

It is possible that there was a high frequency of resistance allele in this field-collected population as it was collected from a region where insecticides were used extensively to manage various maize pests. Similarly, Muraro et al. (2021) [46] evaluated the evolution of resistance to emamectin benzoate in the field-collected population *S. frugiperda*, and after 10 generations of continuous selection to pesticide, the resistance ratio increased ∼2283.44-fold. In previous studies, high resistance levels to indoxacarb have been reported in various lepidopteran insects after continuous selection pressure. For example, *P. xylostella* (2594-fold after six generations of selection) [64], *S. exigua* (240-fold after 12 generations) [32], *H. armigera* (1239-fold after eight generations) [65], and *S. litura* (95-fold after three generations) [66]. In contrast, comparatively low level of indoxacarb resistance has been documented in *H. virescens* (55-fold after six generations) [67], *H. armigera* (4.43-fold after 11 selected generations) [68] and *P. xylostella* (31.3-fold after ten selected generations) [69]. These disparities may be due to differences in species’ geographical origin or to the effects of initial sampling.

The level of dominance resistance in the field depends on several factors, such as the concentration used, the stage of development of the insect, and the environment[42,49]. In the present study, the resistance was characterized as an incompletely recessive trait with polygenic effects when *S. frugiperda* was exposed to indoxacarb. A similar pattern of resistance was reported when *S. litura* [66]*, H. armigera* [68], *S. exigua* [32], and *P. solenopsis* (Tinsley) were exposed to indoxacarb, respectively [70]. In contrast, dominant and polygenic resistance to indoxacarb was reported in *S. litura* [66]. Predictions of effective dominance based on laboratory data, however, must be carefully considered because the range of concentrations required to establish dominance may differ between laboratory and field populations, as well as the effect of inducible insecticidal concentration due to chemical degradation [71]. High levels of cross-resistance between insecticides with the same and different modes of action used in a rotation strategy are one of the key problems for the success of IRM programs. In our study, compared to the Ind-UNSEL strain, the Ind-SEL strain of *S. frugiperda* exhibited obvious cross-resistance to deltamethrin (31.23-fold), low and negligible levels of cross-resistance to chlorfenapyr (3.24-fold), spinosad (2.65-fold), respectively. In previous studies, a low and high level of cross-resistance between indoxacarb, spinosad, flubendiamide, fenvalerate, emamectin benzoate and chlorantraniliprole was found in *S. frugiperda*, *S. exigua, P. xylostella* and *S. litura* [72,73,74,75]. As we know, it has not been reported in other studies for this type of cross-resistance in indoxacarb. It might be that indoxacarb and deltamethrin have the same or cross-molecular targets based on a biochemical mechanism that needs to be investigated.

The oxidative metabolism mediated by cytochrome P450 monooxygenases and the hydrolysis and/or sequestration caused by carboxylesterases is the most frequent mechanisms linked to pesticide resistance in insect pests [76]. Based on the synergistic effects of metabolic inhibitors on indoxacarb toxicity, the involvement of metabolic mechanisms in indoxacarb resistance has been reported in several insect species [34,77]. Present results with synergists showed that the toxicity of indoxacarb against *S. frugiperda* was increased by PBO, indicating that mono-oxygenases P450 enzyme may be associated with indoxacarb resistance in the Ind-SEL population. In a Malaysian field-derived strain of *P. xylostella*, high-level (813-fold) resistance to indoxacarb was greatly reduced by PBO or a PBO analog specific for esterases, suggesting that indoxacarb resistance was attributable to improved metabolic detoxification by esterases [78]. Metabolic resistance associated with an increased level of detoxification enzymes, for example, cytochrome P450, carboxy/cholinesterase (CCE), and glutathione S-transferase (GST)) in insecticide-resistant populations have been reported worldwide [79]. In previous studies, it has been reported that P450, carboxylesterase, and GST were involved in the resistance to indoxacarb in *M. domestica, P. xylostella* but carboxylesterase and GST were the main factors in *S. exigua* leading to indoxacarb resistance [27,78]. Similar to our study, elevated activity of the metabolic enzyme P450 enzyme conferred indoxacarb resistance in *H. armigera* and *S. litura* [68,80]. In a previous study, it was reported that the metabolic inhibitor PBO reduced resistance in the indoxacarb-selected strain, suggesting that metabolic detoxification enzymes were probably involved in indoxacarb resistance in *H. armigera* [34]. These results represent a first step towards understanding the indoxacarb resistance mechanisms in a selected strain of *S. frugiperda*.

The decline in biological fitness among individuals in different insect populations during the development of resistance can influence their relative abundance and genetic impact on future generations. Traits such as insecticide resistance are advantageous when under selection, and genotypes conferring these phenotypes can rapidly increase in a population [81]. Resistance-related fitness costs must be assessed in homozygous resistant individuals and heterozygotes that act as carriers of resistant genes in the early stages of resistance [82]. We evaluated fitness costs in two hybrid populations (Ind-SEL♂ × Ind-UNSEL♀ and Ind-SEL♀ × Ind-UNSEL♂) of the Ind-SEL and the Ind-UNSEL Population. We found significantly longer developmental time of larvae, extended pupal duration, shorter adult longevity, and lower fecundity in the Ind-SEL as compared with the other strains and Ind-UNSEL population. The only parameter that differs between Ind-SEL♂ × Ind-UNSEL♀ (175.68 ± 45.88) and Ind-SEL♀ × Ind-UNSEL♂ (328.03 ± 57.22) was the Net reproductive rate (*R*_0_). On the other hand, all population growth parameters differ between Ind-SEL and Ind-UNSEL strains. Differences in fitness costs associated with insecticide resistance have been reported in many insect populations, including *S. frugiperda*, *H. armigera*, *H. virescens*, *P. xylostella,* and *O. hyalinipennis* [14,66,67,83]. Understanding the occurrence of fitness costs associated with insecticide resistance is essential in developing and implementing IRM programs.

## 5. Conclusions

*S. frugiperda* resistance to indoxacarb has been characterized for the first time in this research and provided data to support resistance management strategies. Results demonstrated that *S. frugiperda* has resistance to indoxacarb and that this can be minimized by rotating this insecticide with chlorantraniliprole, emamectin benzoate, and/or methoxyfenozide due to very low cross-resistance and avoiding rotation with deltamethrin, which has high cross-resistance. Overall, this study highlights the significance of genetics in resistance management strategies and the need for future fitness cost studies to take a more comprehensive approach, as experimental design and criteria may change the results, with significant ramifications for the management of resistant pests in the field.

## Figures and Tables

**Table 1:** LC_50_ values with a resistance ratio of laboratory selection of *S. frugiperda* with indoxacarb in field-collected strains after selection in various generations (G).

Insecticides	LC_50_ (μg-mg L^−1^) (95%CI) ^a^	Slope ± S.E. ^b^	*X*^2^ ^c^	*Df* ^d^	RR ^e^
PHZ19-Pop	0.674 (0.60–0.79)	2.01 ± 0.17	0.063	4	--
Ind-UNSEL(24)	0.427 (0.37–0.48)	2.11 ± 0.168	2.70	4	--
Ind-SEL(G2)	0.787 (0.69–0.90)	1.99 ± 0.171	0.81	4	1.17
Ind-SEL(G3)	0.956 (0.83–1.11)	1.83 ± 0.165	1.79	4	1.42
Ind-SEL(G4)	1.59 (1.38–1.84)	1.84 ± 0.164	0.5	4	2.36
Ind-SEL(G5)	3.20 (2.79–3.66)	2.05 ± 0. 176	2.75	4	4.77
Ind-SEL(G6)	4.08 (3.55–4.54)	2.08 ± 0.18	0.366	4	6.05
Ind-SEL(G7)	5.01 (4.34–5.78)	1.89 ± 0.174	3.32	4	7.43
Ind-SEL(G8)	10.03 (8.68–11.57)	1.97 ± 0.184	3.52	4	14.88
Ind-SEL(G9)	13.68 (11.81–15.81)	1.87 ± 0.171	1.05	4	20.35
Ind-SEL(G10)	15.88 (13.79–18.42)	1.65 ± 0.165	1.69	4	23.56
Ind-SEL(G11)	17.54 (15.14–20.35)	1.77 ± 0.16	1.34	4	26.02
Ind-SEL(G12)	18.84 (16.10–22.08)	1.61 ± 0.15	1.23	4	27.06
Ind-SEL(G13)	19.98 (17.13–23.21)	3.23 ± 0.22	3.25	4	29.64
Ind-SEL(G14)	21.02 (17.96–24.38)	1.88 ± 0.16	1.53	4	31.19
Ind-SEL(G15)	35.06 (29.94–41.54)	3.66 ± 0.23	0.22	4	52.02
Ind-SEL(G16)	50.14 (35.02–73.16)	1.64 ± 0.168	11.40	4	74.39
Ind-SEL(G17)	67.05 (57.09–79.78)	2.49 ± 0.25	3.59	4	99.48
Ind-SEL(G18)	74.03 (65.46–83.17)	2.18 ± 0.23	3.75	4	109.84
Ind-SEL(G19)	87.92 (77.46–99.51)	2.12 ± 0.23	0.81	4	130.45
Ind-SEL(G20)	99.26 (88.01–111.86)	2.28 ± 0.27	0.59	4	147.27
Ind-SEL(G21)	119.88 (105.8–137.85)	2.05 ± 0.24	3.31	4	177.86
Ind-SEL(G22)	137.95 (114.61–163.23)	2.31 ± 0.24	5.36	4	204.67
Ind-SEL(G23)	169.19 (143.52–198.29)	2.34 ± 0.29	4.30	4	251.02
Ind-SEL(G24)	201.83 (169.69–238.39)	2.45 ± 0.32	4.77	4	472.67

^a^ CI = confidence interval, ^b^ S.E. = standard error, ^c^ *X*^2^ = Chi-squared, ^d^ *Df* = degrees of freedom, ^e^ RR = Resistance Ration.

**Table 2:** Concentration mortality (LC; μg mL^−1^) response of *S. frugiperda* strains and crosses to the insecticide indoxacarb after 24 generations of selection.

Insecticides	LC_50_ (μg-mg L^−1^) (95%CI) ^a^	Slope ± S.E. ^b^	*X^2^* ^c^	*Df* ^d^	RR ^e^
Ind-UNSEL	0.427 (0.48–0.37)	2.11 ± 0.168	2.70	4	
Ind-SEL(G24)	201.83 (169.69–238.39)	2.45 ± 2.97	4.77	4	472.67
Ind-res♂ × Sus♀	3.02 (2.42–3.58)	2.98 ± 0.38	6.30	4	7.072
Ind-res♀ × Sus♂	2.58 (1.94–3.28)	2.27 ± 0.21	10.79	5	6.42

LC_50_ values followed by the same letter do not differ significantly due to nonoverlap of 95% confidence intervals (CIs), ^a^ CI = confidence interval, ^b^ S.E. = standard error, ^c^ *X^2^* = Chi-squared, ^d^ *Df* = degrees of freedom, ^e^ RR = Resistance Ratio.

**Table 3:** Susceptibility and cross-resistance of Ind-SEL and Ind-SEL genotypes of *S. frugiperda* to different insecticides.

FAW Genotype Strain	Insecticides	LC_50_ (95% CI) ^a^	Slope (±SE) ^b^	*X*^2^ ^c^ (*df*) ^d^	Resistance Ratio (RR)
Ind-SEL	Deltamethrin	25.3 (20.79–30.86)	1.85 ± 0.172	5.03 (4)	31.23
Indox-UNSEL		0.81 (0.71–0.93)	1.99 ± 0.172	0.24 (4)	---
Ind-SEL	Chlorfenapyr	3.92 (3.18–4.70)	2.16 ± 0.17	5.98 (4)	3.24
ndox-UNSEL		1.21 (1.03–1.42)	1.71 ± 0.18	1.72 (4)	--
Ind-SEL	Chorantraniliprole	1.09 (0.85–1.34)	2.27 ± 0.18	8.35 (4)	1.89
Ind-UNSEL		0.58 (0.51–0.66)	2.02 ± 0.16	2.63 (4)	--
Ind-SEL	Methoxyfenozide	2.32 (1.89–2.79)	2.30 ± 0.20	6.39 (4)	1.59
Ind-UNSEL		1.46 (1.26–1.65)	1.98 ±0.16	1.66 (4)	--
Ind-SEL	Spinosad	1.27 (2.27–2.98)	1.93 ± 0.16	1.27 (4)	2.65
Ind-UNSEL		0.48 (0.41–056)	1.74 ± 0.14	2.75 (4)	---
Ind-SEL	Emamectin-benzoate	0.89 (0.71–1.08)	2.25 ± 0.18	7.23 (4)	1.98
Ind-UNSEL		0.45 (0.37–055)	1.85± 0.15	4.42 (4)	

^a^ CI = confidence interval, ^b^ S.E. = standard error, ^c^ *X^2^* = Chi-squared, ^d^ *Df* = degrees of freedom.

**Table 4:** Concentration–mortality response of *S. frugiperda* larvae exposed to indoxacarb and synergists.

Insecticide	N ^a^	LC_50_ (µg-mg^−1^) (95% CI) ^b^	Slope ± S.E. ^c^	Synergistic Ratio (SR) ^d^
Indoxacarb + UNSEL	630	0.427 (0.37 ± 0.48)	2.11 ± 0.17	---
Indoxacarb + PBO	315	0.366 (0.32 ± 0.42)	2.14 ± 0.17	
Indoxacarb + TPP	315	0.399 (0.35 ± 0.45)	2.06 ± 16	
Indoxacarb + DEM	315	0.451 (0.39 ± 0.51)	2.01 ± 0.16	
Indoxacarb + SEL	630	201.83 (169.69 ± 238.39)	2.35 ± 0.29	---
Indoxacarb + PBO	315	62.53 (54.87 ± 70.85)	1.85 ± 0.17	3.22
Indoxacarb + TPP	315	178.53 (149.57 ± 213.44)	1.91 ± 0.21	1.13
Indoxacarb + DEM	315	195.08 (172.91 ± 219.31)	2.27 ± 0.27	1.04

^a^ N = Number of larvae exposed ^b^ CI = confidence interval, ^c^ S.E. = standard error, ^d^ SR = Synergistic ratio.

**Table 5:** Pre-Adults developmental time (Mean ± SE) of *S. frugiperda*

Parameters	Ind-UNSEL	Ind-SEL♀ × Ind-UNSEL♂	Ind-SEL♂ × Ind-UNSEL♀	Ind-SEL
Egg period (d)	2.94 ± 0.04 ab	3.0 ± 0.00 a	2.98 ± 0.02 a	3.0 ± 0.00 a
1st Instar (d)	2.78 ± 0.06 c	3.17 ± 0.05 b	3.11 ± 0.05 b	3.32 ± 0.07 a
2nd Instar (d)	2.59 ± 0.07 c	3.11 ± 0.05 b	3.00 ± 0.06 b	3.2 ± 0.10 a
3rd Instar (d)	2.77 ± 0.06 c	3.18 ± 0.06 b	3.25 ± 0.05 b	3.21 ±0.06 a
4th Instar (d)	2.59 ± 0.07 c	3.05 ± 0.03 b	3.07 ± 0.04 b	3.51 ± 0.09 a
5th Instar (d)	2.91 ± 0.09 c	3.2 ± 0.06 b	3.05 ± 0.05 c	3.38 ± 0.08 a
6th instar (d)	3.18 ± 0.06 d	3.55 ±0.09 b	3.35 ± 0.08 c	3.66 ± 0.09 a
larval (d)	19.77 ± 0.13 c	22.32 ± 0.13 b	21.81 ± 0.18 b	23.34 ± 0.33 a
Pupal (d)	7.5 ± 0.08 b	7.82 ± 0.16 b	8.8 ± 0.17 a	9.26 ± 0.11 a

The paired bootstrap test at the 5% significance level shows that means followed by the same letters in the same rows are not substantially different. For each treatment, 50 insects were employed.

**Table 6:** Adult longevity (d), APOP, TPOP, Ovi-day, Fecundity, and MGT (Mean ± SE) of *S. frugiperda*.

Parameters	Ind-UNSEL	Ind-SEL♀ × Ind-UNSEL♂	Ind-SEL♂ × Ind-UNSEL♀	Ind-SEL
Adult longevity (d)	10.8 ± 0.48 a	10.2 ± 0.56 b	9.5 ± 0.54 c	9.6 ± 0.7 c
Female longevity (d)	12.9 ± 0.28 a	12.6 ± 0.17 a	11.3 ± 0.3 b	11.6 ± 0.58 b
Male longevity (d)	8.3 ± 0.62 a	7.4 ± 0.76 b	8.6 ± 0.74 a	8.3 ± 0.99 ab
APOP (d)	3.61 ± 0.1 b	4.4 ± 0.13 a	3.13 ± 0.13 bc	4.64 ± 0.44 a
TPOP (d)	30.74 ± 0.18 c	35.45 ± 0.37 b	33.75 ± 0.57 b	37.45 ± 0.56 a
Ovi-day	5.57 ± 0.19 a	5.3 ± 0.18 b	5.08 ± 0.34 b	5.27 ± 0.27 b
Fecundity (eggs/female)	999 ± 55 a	820 ± 17 b	732 ± 53 bc	613 ± 68 c
MGT ^a^	38.05 ± 0.48 c	40.73 ± 0.67 b	40.17 ± 0.66 b	42.26 ± 0.73 a

The paired bootstrap test at the 5% significance level shows that means followed by the same letters in the same rows are not substantially different. For each treatment, 50 insects were employed. ^a^ MGT: Mean generation time.

**Table 7:** Mean generation time, Net reproductive rate, intrinsic rate of increase, and finite rate of increase in *S. frugiperda*.

Parameters	Ind-UNSEL	Ind-SEL♀ × Ind-UNSEL♂	Ind-SEL♂ × Ind-UNSEL♀	Ind-SEL
*r_m_* (day^−1^) ^‡^	0.189 ± 0.005 a	0.150 ± 0.005 b	0.140 ± 0.008 bc	0.123 ± 0.007 c
* *R*_0_	459.66 ± 74.74 a	328.03 ± 57.22 b	175.68 ± 45.88 c	147.15 ± 40.28 c
ƛ (day^−1^) ^§^	1.197 ± 0.006 a	1.162 ± 0.006 b	1.151 ± 0.09 bc	1.131 ± 0.008 c
^†^ *T*	34.1 ± 0.23 c	38.61 ± 0.32 ab	36.74 ± 0.52 b	40.65 ± 0.57 a

The paired bootstrap test at the 5% significance level shows that means followed by the same letters in the same rows are not substantially different. For each treatment, 50 insects were employed. * Net reproductive rate. † Mean length of a generation. ‡ Intrinsic rate of population increase. § Finite rate of population increase.

## Data Availability

The datasets generated and analyzed during this study are available from the corresponding author on request.
